# The autism inpatient collection: methods and preliminary sample description

**DOI:** 10.1186/s13229-015-0054-8

**Published:** 2015-11-10

**Authors:** Matthew Siegel, Kahsi A. Smith, Carla Mazefsky, Robin L. Gabriels, Craig Erickson, Desmond Kaplan, Eric M. Morrow, Logan Wink, Susan L. Santangelo, for the Autism and Developmental Disorders Inpatient Research Collaborative (ADDIRC)

**Affiliations:** Maine Medical Center Research Institute, Spring Harbor Hospital, Tufts University School of Medicine, 123 Andover Road, Westbrook, ME 04092 USA; University of Cincinnati College of Medicine, Cincinnati Children’s Hospital Medical Center, 3333 Burnet Avenue MLC 4002, Cincinnati, OH 45229 USA; University of Colorado School of Medicine, Children’s Hospital Colorado, 13123 E. 16th Avenue, Aurora, CO 80045 USA; University of Maryland School of Medicine, Sheppard Pratt Health System, 6501 N. Charles Street, Baltimore, MD 21204 USA; University of Pittsburgh School of Medicine, 3811 O’Hara St, Pittsburgh, PA 15213 USA; Brown University, Lab for Molecular Medicine, 70 Ship Street, Providence, RI USA; Rhode Island Consortium of Autism Research and Treatment (RI-CART), Developmental Disorders Genetics Research Program, Emma Pendleton Bradley Hospital and Department of Psychiatry and Human Behavior, Alpert Medical School of Brown University, 1011 Veteran Memorial Pkwy, East Providence, RI 02915 USA; Maine Medical Center Research Institute, Tufts University School of Medicine, 509 Forest Avenue, Portland, ME 04101 USA; Maine Medical Center and Maine Medical Center Research Institute, Tufts University School of Medicine, 66 Bramhall Street, Portland, ME 04102 USA

**Keywords:** Autism spectrum disorder, Inpatient, Genetics, Psychiatric, Non-verbal, Intellectual disability, Self-injury, Severe autism, New data resource

## Abstract

**Background:**

Individuals severely affected by autism spectrum disorder (ASD), including those with intellectual disability, expressive language impairment, and/or self-injurious behavior (SIB), are underrepresented in the ASD literature and extant collections of phenotypic and biological data. An understanding of ASD’s etiology and subtypes can only be as complete as the studied samples are representative.

**Methods:**

The Autism Inpatient Collection (AIC) is a multi-site study enrolling children and adolescents with ASD aged 4–20 years admitted to six specialized inpatient psychiatry units. Enrollment began March, 2014, and continues at a rate of over 400 children annually. Measures characterizing adaptive and cognitive functioning, communication, externalizing behaviors, emotion regulation, psychiatric co-morbidity, self-injurious behavior, parent stress, and parent self-efficacy are collected. ASD diagnosis is confirmed by the Autism Diagnostic Observation Schedule – 2 (ADOS-2) and extensive inpatient observation. Biological samples from probands and their biological parents are banked and processed for DNA extraction and creation of lymphoblastoid cell lines.

**Results:**

Sixty-one percent of eligible subjects were enrolled. The first 147 subjects were an average of 12.6 years old (SD 3.42, range 4–20); 26.5 % female; 74.8 % Caucasian, and 81.6 % non-Hispanic/non-Latino. Mean non-verbal intelligence quotient IQ = 70.9 (SD 29.16, range 30–137) and mean adaptive behavior composite score = 55.6 (SD 12.9, range 27–96). A majority of subjects (52.4 %) were non- or minimally verbal. The average Aberrant Behavior Checklist - Irritability Subscale score was 28.6, well above the typical threshold for clinically concerning externalizing behaviors, and 26.5 % of the sample engaged in SIB. Females had more frequent and severe SIB than males.

**Conclusions:**

Preliminary data indicate that the AIC has a rich representation of the portion of the autism spectrum that is understudied and underrepresented in extant data collections. More than half of the sample is non- or minimally verbal, over 40 % have intellectual disability, and over one quarter exhibit SIB. The AIC is a substantial new resource for study of the full autism spectrum, which will augment existing data on higher-functioning cohorts and facilitate the identification of genetic subtypes and novel treatment targets. The AIC investigators welcome collaborations with other investigators, and access to the AIC phenotypic data and biosamples may be requested through the Simons Foundation (www.sfari.org).

## Background

Autism spectrum disorder (ASD) is characterized by a wide range of deficits in social communication and restricted, repetitive behaviors and interests [1]. It has recently been noted that those most severely affected by ASD, including those with intellectual disability, expressive language impairment (non- or minimally verbal), low adaptive functioning, and/or externalizing behaviors, have been understudied [2]. Severely affected individuals with ASD are underrepresented in extant large collections of phenotypic and genomic data available to investigators, which may limit the field’s understanding of ASD etiology, and certainly has a negative impact on the ability to effectively treat these patients. These knowledge gaps are also of great clinical concern, as communicative and cognitive abilities are the best predictors of long-term outcomes in children with ASD [3, 4]. Developing a research platform that can systematically assess large numbers of severely affected individuals may accelerate the pace of ASD etiological research and provide unique opportunities to identify ASD subtypes and test targeted treatments.

Barriers to the study of children who are more severely affected by ASD include challenges in recruitment in outpatient settings and the relative lack of measures that have been validated for use with non-verbal or intellectually disabled individuals with ASD. In addition, it may be easier for investigators to perform some research procedures, such as phlebotomy, neuroimaging, and physiologic measurements, with a more able sample. When faced with a population difficult to study in the outpatient setting, the National Institute of Mental Health has previously employed inpatient research units to perform intensive characterization of other complex disorders, such as childhood onset schizophrenia [5].

To address the paucity of research on this segment of the ASD population, the authors formed the Autism and Developmental Disorders Inpatient Research Collaborative (ADDIRC), a network of six specialized hospital psychiatry units in the United States, and engaged with several autism philanthropic foundations to explore opportunities to leverage our unique resources and access to these children. Prior survey data indicated a high volume of patients in specialized inpatient units likely had ASD with intellectual disability, expressive language impairment, and/or externalizing problem behaviors [6]. When considering how to systematically assess and leverage this population, we noted the success of the Simons Simplex Collection (SSC), which produced a searchable database of phenotypic data and corresponding biological samples from more than 2700 autism families, contributed to large gene sequencing efforts and spawned over 100 publications. The SSC was conducted with outpatients who were relatively high functioning and predominantly male (mean non-verbal intelligence quotient (IQ) = 86.5, Vineland Adaptive Behavior Scale standard score = 74.0, and 85 % male) [7]. Capitalizing on the unique population and resources of the inpatient setting, and informed by the experience of the Simons Foundation in creating the SSC, we initiated the Autism Inpatient Collection (AIC) to provide a resource for study of the full autism spectrum. Our long-term goals include contributing to efforts to assemble cohorts with rare mutations, copy number variants, and other chromosomal anomalies that are large enough for meaningful description and analysis, in order to accelerate the identification of autism subtypes and targeted treatments.

The goals of this preliminary study were to test the feasibility of using the specialized inpatient setting to perform systematic autism research and to begin to characterize the population of children and adolescents with ASD who are admitted for inpatient psychiatric care. We expected that the AIC would contain a relatively high proportion of individuals with autism and intellectual disability, female gender, low adaptive functioning, and/or minimal to non-verbal language status, and that the inpatient setting might facilitate high rates of enrollment. Here we describe the preliminary characteristics of the first 147 subjects in the AIC cohort and methods used to obtain the data.

## Methods

The AIC study is being conducted in six academically affiliated specialized child psychiatry hospital units: Bradley Hospital (Brown University; RI), Cincinnati Children’s Hospital (University of Cincinnati; OH*),* Children’s Hospital Colorado (University of Colorado, CO), Sheppard Pratt Health Systems (University of Maryland, MD), Western Psychiatric Institute and Clinics (University of Pittsburgh, PA), and Spring Harbor Hospital/Maine Medical Center Research Institute (Tufts University; ME), which also serves as the AIC coordinating site with data and analytic cores. The units specialize in the assessment and treatment of children with ASD and other developmental disorders. The programs utilize a multi-disciplinary bio-behavioral approach that substantially differs from standard inpatient child psychiatric care [8]. Children are typically admitted due to externalizing problem behaviors (aggression, self-injury, or tantrums) [9, 10], and admissions are funded by public and private health insurance.

The study protocol and standard operating procedures were developed through a series of teleconferences with participating sites and an in-person study launch meeting. Research assistants underwent a 2-day training at the coordinating site on informed consent administration, data collection, and data security procedures and receive continued support via weekly calls with the project manager to review study procedures, compliance, and best practices. Study site visits were conducted by the coordinating site after initiation of data collection to review protocol fidelity, regulatory compliance, and documentation of informed consent. A scientific advisory group and coordinating site advisory group provide guidance on scientific and administrative issues, and an interactive relationship with the funding organizations leverages their prior experience and expertise in assembling large collections.

### Patient recruitment

Children admitted to the hospital units were screened for study eligibility using the Social Communication Questionnaire [11]. Children with a score of ≥12 on the SCQ, or who scored less than 12 but were referred by the inpatient team due to high suspicion of ASD, were eligible for enrollment. Subjects who met inclusion criteria and whose legal guardian provided informed consent within 5 days of admission were offered enrollment in the study. Inclusion criteria were as follows: age 4 to 20 years, not having “prisoner” status (on probation or house arrest), and having at least one guardian who spoke and read English proficiently and who knew the child at 4–5 years of age.

The informed consent form requested permission to re-contact families for future studies, and participants were asked to enroll in the Autism Inpatient Community at the Interactive Autism Network (AIC@IAN), an online community that will facilitate dissemination of study results and re-contacting of the study cohort. Permissions for collection, storage, and future access to biosamples (blood, saliva, plasma) were also requested, utilizing the NIH-recommended language for genetic studies of subject biomaterials. Consent was also obtained for transfer of de-identified data to a searchable online data portal (SFARI Base) and the National Database for Autism Research (NDAR). To protect the privacy of subjects, and to facilitate the linking of phenotypic and biological data, a global unique identifier (GUID) [12] was assigned to each subject and family member. The study protocol was approved by the Institutional Review Board at each of the six investigating sites: the Cincinnati Children’s Hospital Medical Center Human Research Program, the Lifespan – Rhode Island Hospital Research Protection Office, the Maine Medical Center Office of Research Compliance, the Sheppard Pratt Institutional Review Board, the University of Pittsburgh Research Conduct and Compliance Office, and the University of Colorado Denver Office of Regulatory Compliance.

### Evaluation of probands and families

The diagnosis of ASD was made by a research-reliable Autism Diagnostic Observation Scale – 2 (ADOS-2) [13] examiner using DSM 5 criteria, including ADOS-2 results in addition to a review of study measures and inpatient observation. Diagnosis of ASD required meeting the empirically derived cutoffs for autism or autism spectrum disorder on the ADOS-2. Clinical assessment, using all available data, indicating that a child did not have ASD could override a positive ADOS-2. Developmentally delayed children who screened positive with an SCQ score of ≥12 and were enrolled in the study, but who were found to not have ASD, will ultimately serve as a small non-ASD comparison population. ADOS-2 examiners, who were master’s or doctoral level clinicians with extensive experience evaluating children with ASD, underwent a 2-day initial research reliability training session with a certified ADOS-2 trainer (RLG), and then achieved at least 80 % inter-rater reliability with the trainer on two consecutive ADOS-2 administrations of each module set (Modules 1 and 2; Modules 3 and 4) per current research reliability standards established by the test authors [13]. To minimize administration and coding drift, monthly calls were held with the ADOS-2 examiners and trainer, and a 2-day recalibration meeting was held 6 months after study initiation.

An assessment battery was developed with a focus on domains most relevant to this population and measures appropriate for use in those with intellectual disability or non-verbal status (see Table 1). A primary caregiver provided demographic information, family composition, proband and family medical and psychiatric history, and the proband medication profile. The caregiver was then administered the Aberrant Behavior Checklist – Community (ABC-C) [14], Repetitive Behavior Scale – Revised – Self-Injury Subscale [15], Child and Adolescent Symptom Inventory 5 [16], Vineland-2 parent report form [17], Parent Stress Index – 4 – Short Form [18], Difficult Behavior Self-Efficacy Scale (DBSES) [19], and a newly developed measure – the Emotion Dysregulation Index (EDI). The EDI is a treatment-sensitive outcome measure of emotional distress and problems with emotion regulation designed to be valid for the full range of cognitive and communication abilities in children with autism (R01HD079512) [20]. The EDI was repeated at discharge, and the ABC-C – Irritability and Hyperactivity Subscales, PSI-4-SF, DBSES, and medication profile were repeated at discharge and by telephone at 2-month follow-up. Self-injurious behavior (SIB) was also evaluated by administration of the Functional Assessment Screening Tool [21] to the caregiver and an inpatient staff member, and a final determination of the presence of clinically significant SIB and its function was made by the unit behavioral specialist (psychologist or board certified behavior analyst (BCBA)), based on all available information. Axis 1 co-morbid psychiatric disorders and Axis 3 medical conditions were recorded at discharge.

**Table 1:** AIC study measures

Measure	Domain/construct	Format
ADOS-2	Diagnostic confirmation; repetitive behaviors and social communication	Structured assessment
Social Communication Questionnaire (SCQ)	Diagnostic screen; repetitive behaviors and social communication	Parent report
Aberrant Behavior Checklist (ABC)	Challenging behaviors	Parent report
Repetitive Behavior Scale Revised (RBS-R) – Self-Injury Subscale	Self-injurious behavior	Parent report
Functional Assessment Screening Tool (FAST)	Behavioral function for self-injurious behavior	Parent and observer report
Vineland-2	Adaptive functioning and communication	Parent report
Difficult Behavior Self-Efficacy Scale (DBSES)	Self-efficacy	Parent report
Leiter 3	Cognitive ability	Structured assessment
Child and Adolescent Symptom Inventory (CASI-5)	Psychiatric symptoms and disorder cutoffs	Parent report
Parent Stress Index	Parenting stress	Parent report
Emotion Dysregulation Inventory (EDI)	Emotional distress and problems with emotion regulation	Parent report

### Phenotypic and biologic data collection and curation

Phenotypic data were collected on paper forms and entered at each site into a web-based, HIPPA-compliant central database through the Research Electronic Data Capture (REDCap) system. The REDCap database was built with over 100 logic checks to detect and reduce data entry errors and is monitored by the study data coordinator. Whole blood was collected from the proband and biological parents in one EDTA vacutainer tube and two sodium citrate tubes. If blood could not be obtained, a saliva sample was collected using an Oragene-DNA (OGR-500) 2-ml saliva collection tube. Samples were shipped to the Rutgers University Cell and DNA Repository (RUCDR), where DNA was extracted from blood or saliva, plasma was stored, and lymphoblastoid cell lines were created. Phenotypic and biological data were linked by a GUID. The phenotypic data will be made available by the Simons Foundation through an interactive platform, SFARI Base, and access to both the phenotypic data and biological samples can be requested from the Simons Foundation by qualified investigators. After primary analyses are completed, phenotypic data will also be sent to the National Database for Autism Research (NDAR). In addition, we are collaborating with the Autism Sequencing Consortium (ASC), and whole exome sequencing data on these samples will be released to the database of Genotypes and Phenotypes (dbGaP) by the ASC as they become available.

### Analysis

Descriptive statistics were computed using means and standard deviations for continuous variables (Medians (*Md*) for non-parametric data), and frequencies and proportions for categorical variables. To examine potential gender differences, we conducted Independent Samples *T* tests (Mann-Whitney *U* tests for non-parametric data) or chi-square tests for continuous and categorical data, respectively. Statistical significance is indicated by *p* values ≤0.05.

Intellectual disability was defined as a non-verbal IQ of ≤70, low adaptive functioning was defined as a Vineland Adaptive Behavior Scale – 2 (VABS-2) score ≤70, non-verbal and minimally verbal status was defined as use of an ADOS-2 Module 1 or 2, and the presence of clinically significant externalizing problem behaviors was defined as an Aberrant Behavior Checklist – Irritability Subscale score of ≥16 [22]. Self-injurious behavior was defined as the presence of at least daily attempts at self-injury, as determined by the unit psychologist or board certified behavior analyst.

## Results

**Fig. 1:**
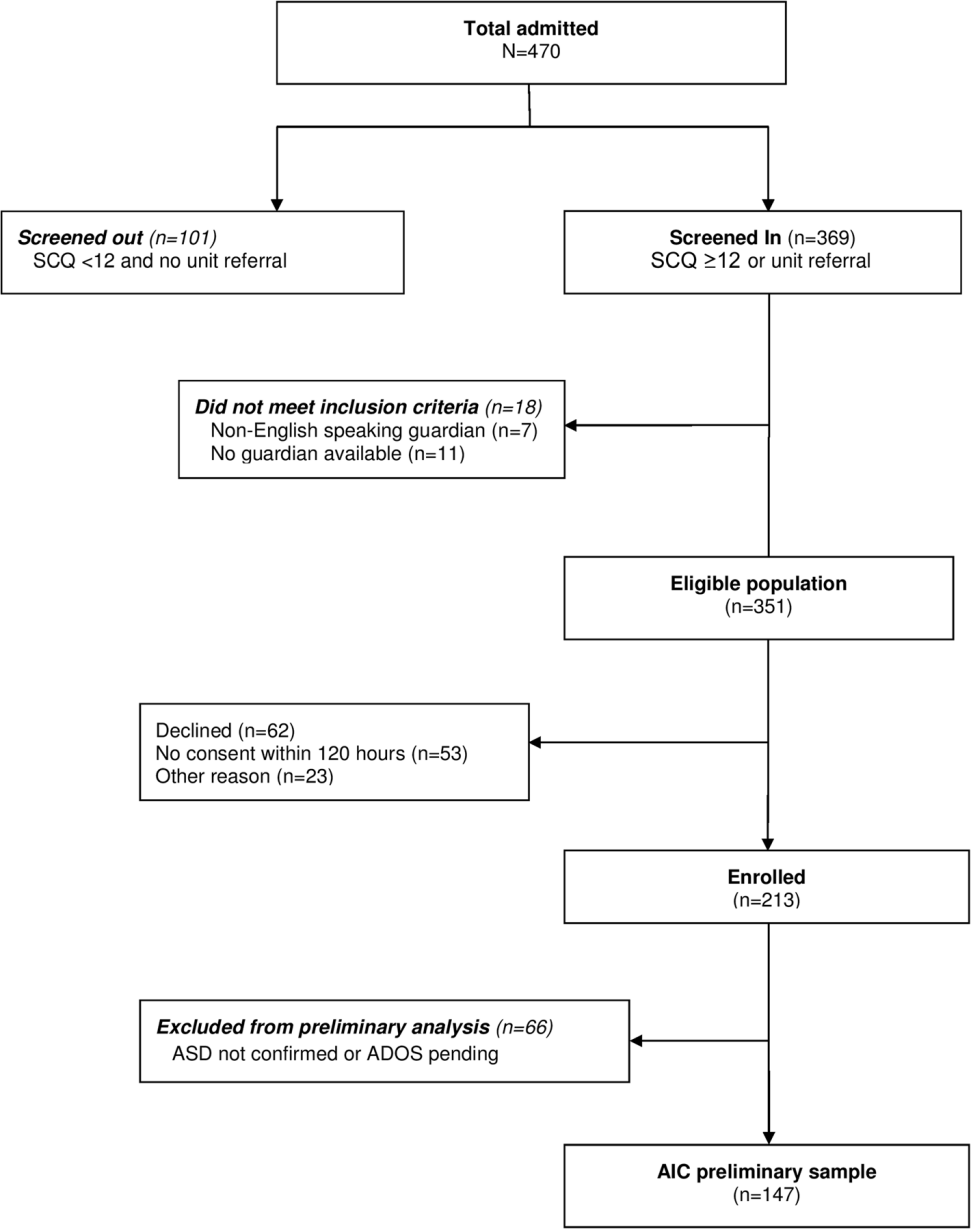
Autism inpatient collection preliminary sample

Of the first 470 children screened for study eligibility, 351 met inclusion criteria and 213 (61 %) were enrolled (see Fig. 1). One hundred forty-seven enrolled subjects (69 %) were confirmed to have a diagnosis of ASD at the time of this preliminary analysis. The sample was 26.5 % female, had a mean age of 12.6 years, and was 74.8 % Caucasian and 81.6 % non-Hispanic/non-Latino (see Table 1).

As expected, a sizable proportion of the study sample had intellectual disability (ID) (42.6 %), expressive language impairment (52.4 % non- or minimally verbal), low adaptive functioning (71.9 %), and/or externalizing problem behaviors (85.4 % ≥16 on ABC – Irritability Subscale). Mean non-verbal IQ for the sample was 70.86 (SD 29.16, range 30–137) as measured on the Leiter 3, and the mean Adaptive Behavior Composite on the VABS-2 was in the extremely low range (M 55.57, SD 12.88, range 27–96). The average VABS-2 Expressive Communication subscale standard score was 6.55 (SD 3.92, range 1–17), which is almost 3 standard deviations below the population mean of 15 [17]. Fifty-two percent of the sample was non-verbal or minimally verbal. Self-injurious behavior was present in 26.5 % of the sample, and the mean score on the Aberrant Behavior Checklist – Irritability Subscale was 28.6 (SD 8.6, range 8–45), well above the typical entry criteria score of 16 for treatment studies targeting externalizing problem behaviors.

**Fig. 2:**
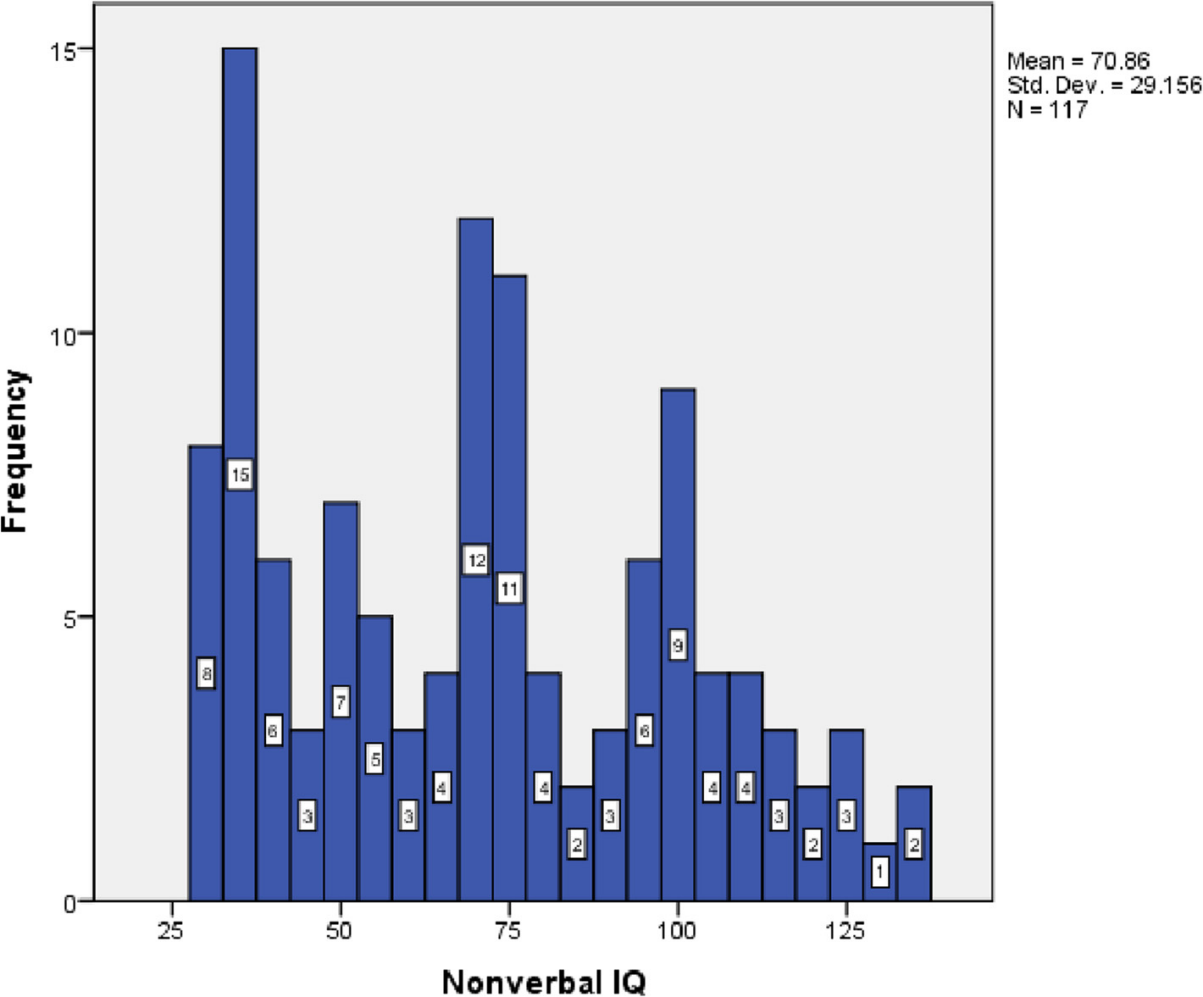
Non-verbal IQ distribution

Female subjects had a lower mean non-verbal IQ and a notably higher rate of self-injurious behavior (SIB) (33 %), compared to males (24 %) (Fig. 2). The frequency and severity of SIB, as measured by the RBS-R – Self-Injury Subscale score, was significantly higher for females in both the total sample (females *Md* = 10.0, *n* = 35, males *Md* = 7.0, *n* = 93, *Mann-Whitney U* = 1113.50, *z* = −2.75, *p* = .006, *r* = 0.24) and in those with clinically significant SIB (females *Md* = 15.0, *n* = 13, males *Md* = 9.0, *n* = 26, *Mann-Whitney U* = 77.50, *z* = −2.73, *p* = .006, *r* = 0.44) (Table 2).

**Table 2:** Sample characteristics

	Overall sample		Males		Females	
	(*n* = 147)		(*n* = 108)		(*n* = 39)	
	Mean (SD)/*N* (%)	Range	Mean (SD)/*N* (%)	Range	Mean (SD)/*N* (%)	Range
Age (years)	12.58 (3.42)	4–20	12.55 (3.55)	4–20	12.67 (3.07)	6–18
Ethnicity (non-Hispanic/Latino)	120 (81.6 %)		86 (79.6 %)		34 (87.2 %)	
Race (White)	110 (74.8 %)		83 (76.9 %)		27 (69.2 %)	
Non-verbal IQ (*n* = 117)	70.86 (29.16)	30–137	73.01 (29.84)	30–137	64.63 (25.56)	31–113
Expressive Communication Subscale (Vineland-2)	6.55 (3.92)	1–17	6.65 (4.05)	1–17	6.26 (3.53)	1–17
Adaptive Behavior Composite (Vineland-2) (*n* = 128)	55.57 (12.88)	27–96	55.33 (12.79)	27–96	56.27 (13.36)	29–78
ADOS-2 Module administered						
1	62 (42.2 %)		46 (42.6 %)		16 (41 %)	
2	15 (10.2 %)		9 (8.3 %)		6 (15.4 %)	
3	57 (38.8 %)		43 (39.8 %)		14 (35.9 %)	
4	13 (8.8 %)		10 (9.3 %)		3 (7.7 %)	
ADOS-2 scores: Modules 1, 2, and 3 only (*n* = 134)						
Comparison score	7.47 (1.57)	4–10	7.52 (1.55)	4–10	7.36 (1.64)	4–10
Social affect	13.44 (4.26)	2–20	13.4 (4.23)	2–20	13.56 (4.16)	5–20
Restricted/repetitive behaviors	3.44 (2.12)	0–8	3.50 (2.11)	0–8	3.28 (2.16)	0–7
ADOS-2 scores: Module 4 only (*n* = 13)						
Social and communication	12.31 (3.40)	8–19	12.0 (2.87)	8–16	13.33 (5.51)	8–19
Stereotyped behaviors and restricted interests	2.92 (3.23)	0–13	3.20 (3.55)	1–13	2.0 (2.0)	0–4
Aberrant Behavior Checklist (ABC) Subscales						
Irritability	28.58 (8.6)	8–45	27.72 (8.5)	8–45	31.15 (8.2)	10–42
Lethargy	15.98 (8.5)	1–44	15.99 (8.6)	1–44	15.94 (8.3)	1–39
Stereotypy	8.98 (5.7)	0–20	8.62 (5.6)	0–20	10.06 (5.9)	2–20
Hyperactivity	30.58 (10.2)	1–47	30.73 (10.4)	1–47	30.15 (9.7)	11–45
Inappropriate speech	5.61 (3.8)	0–12	5.61 (3.8)	0–12	7.03 (3.6)	0–12
Self-injurious behavior (SIB) present	39 (26.5 %)		26 (24.1 %)		13 (33.3 %)	
Repetitive Behavior Scale – Revised (RBS-R) – Self-Injury Subscale total score for subjects with SIB present (*n* = 39)	*n* = 39		*n* = 26		*n* = 13	
	Md = 11.0		Md = 9.0^a^		Md = 15.0^a^	

*Md* median

^a^Significant difference between male and female RBS-R scores, Mann-Whitney *U* = 1113.50, *z* = −2.75, *p* = 0.006

## Discussion

Preliminary results from the first 147 subjects with ASD enrolled in the Autism Inpatient Collection (AIC) support the feasibility of utilizing the specialized inpatient psychiatric unit setting to study complex disorders with variable presentations, and the opportunity to capture the full range of ASD severity. Enrollment of 61 % of eligible subjects within 5 days of admission, in the context of an intensive assessment protocol that includes a blood draw, suggests that the inpatient setting is an efficient means of recruiting and assessing research participants with ASD, including those who may be difficult to reach or assess in the outpatient setting. The AIC cohort is notable in its average severity of impairment across a number of phenotypic domains including cognitive ability, adaptive functioning, expressive language, and externalizing problem behaviors. Simultaneously, the cohort captures the full range of ASD severity, as reflected by scores that range from severe impairment to superior functioning.

Over one fourth (26.5 %) of the sample is female, which is higher than the rate of 22.5 % reported by the Centers for Disease Control [23] and higher than other comparable large-scale ASD collections, such as the SSC which is 15.4 % female [7]. The higher rate of females may be attributed to the fact that our sample is weighted toward the more severely affected, as the prevalence of ASD in females is greater among those with severe cognitive impairment [24]. The largest study of gender differences in ASD to date found that females had significantly higher rates of both general externalizing problems and SIB than males [25]. In the AIC sample, approximately one third of females were observed to engage in daily SIB and they had significantly greater frequency and severity of SIB than males. Given the relatively high proportion of females and prevalence of SIB in our sample, the AIC data offer an opportunity to better describe and understand the female autism phenotype.

The frequency of co-occurrence of intellectual disability (ID) in individuals with ASD has varied substantially across studies and over time. Most current epidemiologic studies cite a prevalence of ID of 20–40 % [23, 26]. Taking a conservative approach, we utilized the Leiter 3 non-verbal IQ test in order to provide the greatest chance for even very impaired subjects to provide a reliable estimate of their cognitive ability. Despite this, nearly half the sample scored in the ID range, and almost three quarters had extremely low adaptive functioning. These numbers may still underestimate the severity of intellectual disability in the cohort, as 11 subjects (7.5 %) were unable to perform the Leiter tasks, suggesting they may have a mental age of less than 3 years, which is below the floor for the Leiter test. Greater degrees of intellectual disability in ASD have been associated with decreased expressive language, increased rates of self-injury, and specific genetic anomalies. As the cohort grows, interrogating the genomes of AIC participants may shed light on the specific and synergistic contributions of polymorphisms and mutations associated with ASD and intellectual disability.

The AIC sample also presents an opportunity to investigate individuals with significant expressive language impairment, an area identified as under-researched by the Inter-Agency Autism Coordinating Committee of the NIH [2]. Over half of the AIC sample is non-verbal or minimally verbal. This most likely represents a stable language status, rather than a pre-verbal condition, as the mean age of the AIC is 12.6 years, almost all subjects are older than 5 years, and the likelihood of a substantial change in verbal status (from non- or minimally verbal to verbally fluent) decreases dramatically after age 6 [27]. Communication is one of the two core deficits in autism, but research and treatment of individuals with ASD and serious expressive language impairment has been slowed by a lack of gold standard research methods for characterizing verbal status or communication ability, great heterogeneity in the use of clinical measures, and the absence of a consistent definition of non-verbal status [28]. Very little is known, in particular, about school-age children with ASD who are non-verbal or minimally verbal. As over 50 % of the AIC sample is in this category, this may represent a research opportunity for this often neglected segment of children with ASD.

Externalizing problem behaviors are often the primary reasons for children with ASD to present for treatment in all settings, not just to inpatient units [29], and are highly predictive of caregiver stress [30]. The AIC sample includes a high proportion of children with these behaviors, a group that is often excluded or unable to participate in outpatient studies, particularly large-scale phenotyping studies with multiple assessments. Externalizing problem behaviors in ASD are typically measured by the Aberrant Behavior Checklist – Irritability Subscale, which captures physical aggression, self-injurious behavior (SIB), and tantrums. In the AIC sample, the mean ABC-I score was 28.6, which is similar to the pre-treatment score of ASD samples recruited for antipsychotic medication clinical trials [22, 31], indicating that the average AIC subject has serious behavioral disturbance. Moving beyond the parent-reported ABC-I, the size of the AIC sample and range of measures collected offers the opportunity to examine factors that may underlie these behaviors, particularly through the use of latent-class multi-level modeling.

Self-injurious behavior, in particular, is prevalent in the sample and is an under-recognized source of impairment in ASD. SIB has been shown to occur at twice the rate of physical aggression in a community ASD sample [32]. Despite its prevalence, the conceptualization and measurement of SIB varies by reporter and environment [33]. SIB is also the subject of few large studies, primarily receiving attention in single-case designs in the applied behavioral analysis literature. Not surprisingly, evidence for SIB treatment options other than individualized behavioral analysis is minimal, and includes no proven pharmacologic treatments. The size of the AIC sample exhibiting SIB as well as the behavioral expertise and opportunity for 24-h observation on the inpatient units offer the possibility for more refined measurement and novel mechanistic, physiologic, and genetic investigations of SIB. For example, the combined contributions of arousal, cognitive control, and emotional dysregulation have been proposed as a trans-diagnostic paradigm for understanding negative externalizing behaviors in autism [34], and may be ideal to investigate in the laboratory-like setting of the hospital unit.

Looking ahead, we continue to expand the size of the AIC cohort in order to provide a sample size adequate for phenotype–genotype explorations for autism subtypes. We also seek to capitalize on the availability of large numbers of individuals with intellectual disability, expressive language impairment, female gender, and SIB in order to better understand the challenges, needs, and treatment options of these subgroups. Efforts have already begun to include the AIC biosamples in current large genomic sequencing efforts, such as the Autism Sequencing Consortium (ASC) [35]. This collaboration is anticipated to both facilitate access to the AIC genomic data and to enhance the value of the larger ASC effort by including more of those who are severely affected.

The investigators of the AIC welcome collaborations with other interested investigators. Access to the AIC phenotypic and genetic data and biosamples can be requested through the Simons Foundation. The data will be made available through an interactive platform, SFARI Base, and the phenotypic data will also be sent to the National Database for Autism Research (NDAR). Whole exome sequencing data on these samples will be released to dbGaP by the Autism Sequencing Consortium as they become available.

### Limitations

Results presented here are preliminary, based upon the first 147 subjects in the AIC; characteristics of the full AIC sample may vary somewhat. Although the control group is not yet large enough for meaningful comparison, we will, in future reports, include a statistical comparison with a comparison group of individuals with non-ASD developmental delay that are enrolled but fail to meet criteria for ASD. Finally, although the strength of the AIC is the inclusion of individuals typically underrepresented in outpatient studies, results may be limited in their generalizability to the broader autism population as the collection is composed of children who have been hospitalized due to externalizing problem behaviors.

## Conclusions

Preliminary results indicate that the Autism Inpatient Collection may be a valuable cohort for ASD research. The sample is older and has a higher proportion of females, lower intellectual and adaptive functioning, greater expressive language impairment, and higher rates of self-injury and other externalizing problem behaviors than most other large collections currently available to investigators. The availability of these data, and corresponding biosamples and genomic data, through the Simons Foundation and NDAR, may accelerate the identification of genetically distinct autism subtypes and provide opportunities for re-contacting of cohorts to identify unmet medical needs and potential treatment targets.

## Abbreviations

ABC: Aberrant Behavior Checklist
ABC-I: Aberrant Behavior Checklist – Irritability Subscale
ADDIRC: Autism and Developmental Disorders Inpatient Research Collaborative
ADOS-2: Autism Diagnostic Observation Schedule – 2
AIC: Autism Inpatient Collection
AIC@IAN: Autism Inpatient Community at the Interactive Autism Network
ASC: Autism Sequencing Consortium
ASD: autism spectrum disorder
BCBA: board certified behavior analyst
dbGaP: database of Genotypes and Phenotypes
DBSES: Difficult Behavior Self-Efficacy Scale
EDI: Emotion Dysregulation Inventory
GUID: global unique identifier
ID: intellectual disability
IQ: intelligence quotient
NDAR: National Database for Autism Research
PSI-4-SF: Parent Stress Index – 4 – Short Form
RBS-R: Repetitive Behavior Scale – Revised
REDCap: Research Electronic Data Capture
RUCDR: Rutgers University Cell and DNA Repository
SCQ: Social Communication Questionnaire
SIB: self-injurious behavior
SSC: Simons Simplex Collection
VABS-2: Vineland Adaptive Behavior Scale – 2
